# Rethinking Oral Health in Aging: Ecosocial Theory and Intersectionality

**DOI:** 10.1177/00220345231175061

**Published:** 2023-06-14

**Authors:** L. Slack-Smith, T. Ng, M.E. Macdonald, A. Durey

**Affiliations:** 1 School of Population and Global Health, University of Western Australia, Perth, Australia; 2 Department of Medicine, Dalhousie University, Halifax, Canada

**Keywords:** intersectional framework, social isolation, healthcare disparities, social determinants of health, research methodology, social inequity

## Abstract

Poor oral health affects the health and well-being of older adults in many ways. Despite years of international research investigating poor oral health among older adults, it has remained a largely unresolved problem. The aim of this article is to explore the combination of 2 key frameworks, ecosocial theory and intersectionality, to guide our exploration and understanding of oral health and aging and help inform research, education, policy, and services. Proposed by Krieger, ecosocial theory is concerned with the symbiotic relationship among embodied biological processes and social, historical, and political contexts. Building on the work of Crenshaw, intersectionality explores how social identities such as race, gender, socioeconomic status, and age interconnect in ways that can enhance privilege or compound discrimination and social disadvantage. Intersectionality offers a layered understanding of how power relations reflected in systems of privilege or oppression influence an individual’s multiple intersecting social identities. Understanding this complexity and the symbiotic relationships offers an opportunity to reconsider how inequities in oral health for older adults can be addressed in research, education, and practice and increase the focus on equity, prevention, interdisciplinary care, and use of innovative technology.

## Introduction

Rousseau et al. (2014) captured how the stigma of compromised oral health in aging, the grief around tooth loss, and the shame of wearing dentures can greatly affect an individual’s well-being. These issues interface with agism in industrialized countries, reflecting negative and often discriminatory views about older people, compounded by issues such as racism and sexism directed at people in minority groups. They noted, “Future research could usefully explore the ways in which older people’s experiences and expectations of oral health and function of the mouth are shaped by the cohort(s) to which they belong” (p. 472). For example, an aging Indigenous woman living in a rural area without fluoridated water will have experiences that may overlap with, but are also distinct from, an urban immigrant man who has retired on social assistance; in both scenarios, their contexts will affect their oral health outcomes. People’s lives are complex; however, research, professional education, policy, and health services do not frequently recognize and respond to this complexity, instead adopting a one-size-fits-all approach. MacEntee et al. (1997) called for new models to consider oral health in aging.

Despite international research over many years, oral health among older adults has remained a largely unresolved “wicked” problem (Barrett 1998; McKenna et al. 2020). With aging populations on the rise in industrialized societies, this is cause for concern (Thomson and Ma 2014; McKenna et al. 2020). While using the term “wicked problem” seems fitting in this scenario, it must not mask how power and privilege create differentials (Alford and Head 2017; Turnbull and Hoppe 2019).

Although a focus on individuals and their lived experiences and perceptions is important in research and practice for oral health in older adults, acknowledging the role of societal and structural issues is also needed, moving away from the restrictive focus on personal responsibility and individual risk factors (Newton and Bower 2005; Watt 2007). Slow progress in addressing this seemingly wicked problem so that older adults’ oral health outcomes improve leads us to consider the lens through which we view oral health. Cuesta and Rämgård (2016) identify how various elements intersect to either facilitate or inhibit change, and they highlight the importance of power relations where elements such as gender and ethnicity intersect with processes of power in ways that can enable or compromise care.

To investigate this wicked problem, we bring together 2 theoretical frameworks, each of which focuses on macrolevel causes and power (Merz et al. 2021) but neither has been sufficiently integrated into oral health research: ecosocial theory and intersectionality. Ecosocial theory is concerned with the symbiotic relationship among embodied biological processes and social, historical, and political contexts—that is, how ill health and health inequalities are produced and reproduced through social structures such as poverty. Ecosocial theory allows us to take the insights and apply them to a theory of disease causation (Krieger 1994, 2020). Intersectionality invites us to consider how “upstream social determinants such as racism, sexism and classism form interlocking systems of oppression that shape the experiences and life chances of individuals as a consequence of their multi-dimensional social identities” (Green et al. 2017, p. 214).

## Oral Health in Older Adults

Importantly, with the older adult population increasing in number and proportion, beliefs are changing that tooth loss and poor oral health are a natural consequence of normal aging (Slade et al. 2014; Bassim 2018). However, with aging can come reduced mobility and cognitive function and declining financial security and access to healthy nutrition, resulting in the increased need for dental services yet with difficulties accessing them (e.g., transportation, financing; Bassim 2018; Kotronia et al. 2021). Evidence suggests that poor oral hygiene is a strong predictor of dental caries and periodontal disease—both of which are considered preventable (Kossioni et al. 2018). In addition, periodontal disease (and tooth loss) has been linked to comorbidities such as cardiovascular disease, diabetes, and respiratory disease (Kossioni et al. 2018), all of which have significant health consequences for older adults.

Yeung (2018) suggests that oral health care systems require an intersectoral and interprofessional approach to adapt to the changing needs of older adults. Such an approach includes dental professionals, policy makers, health and medical professionals, public health professionals, and researchers working together rather than in siloes and integrating their knowledge for better oral health outcomes. In addition, Yeung (2018) suggests a plan of action that includes oral care in primary health care, promotes oral health across the life course, and informs evidence-based oral health policies. Yeung also notes financial barriers to accessing oral care.

Public health dentistry provides a useful lens for thinking about oral health in older adults. Using a public health approach, we can divide the drivers and consequences of oral health as follows:

*Downstream*: Oral disease is one of the most common and costly diseases of the life course, leading to pain, infections, tooth loss, lost productivity, chronic medical conditions, delayed growth, and cognitive development by interfering with nutrition, concentration, and community participation (Selwitz et al. 2007). Globally, its substantial impact is reflected in morbidity and cost (Righolt et al. 2018).
*Upstream*: Little is being done upstream to change these downstream outcomes. Focus is on treatment rather than prevention; costs are prohibitive in many settings with limited attention given to oral care in primary health care, including that in residential aged care (Cohen et al. 2017; Slack-Smith et al. 2023).

The current implementation of the social determinants of health still largely focuses on a biomedical, individual, and depoliticized approaches (Bolte and Lahn 2015). The results include making inequities visible yet lacking the “comprehensive theorization of the complexity of societal causes” and shying from calls to redistribute wealth and power or critique neoliberalism with its focus on individual responsibility (Green 2010). To really understand and respond to the oral health needs of older adults, attention must be given to how individual pathology is embedded in socioeconomic context, with a focus on how they intersect to affect oral health outcomes and overall quality of life (Bolte and Lahn 2015; Green 2010).

## Ecosocial Theory

To further delve into the issues of power and marginalization and develop ideas of intersectionality, we draw on the ecosocial theory. Krieger (2020) proposed ecosocial theory in 1994 as an epidemiologic theory of disease causation, which “conceptualizes health inequities in relation to power, levels, life-course, historical generation, biology, and ecosystems” (p. 45). Ecosocial theory comprises 4 constructs: “(a) embodiment; (b) pathways of embodiment; (c) cumulative interplay of exposure, susceptibility, and resistance across the life-course and across levels; and (d) accountability and agency” (p. 45). Put simply, ecosocial theory is concerned with the symbiotic relationships among embodied biological processes and social, historical, and political contexts—that is, how ill health and health inequalities are produced and reproduced through social structures (Krieger 2011). It is concurrently focused on exposure, susceptibility, and resistance (Krieger 2012).

## Intersectionality Framework

Adding to ecosocial theory, intersectionality provides a framework that theorizes complexity across the multiple social identities that people live. While ecosocial theory is a theory of causation, intersectionality is more an analytic perspective with a focus on identity and power (Merz et al. 2021). Intersectionality, popular in qualitative approaches, can increase our understanding of how multiple sociocultural elements create identities and inform perceptions and experiences associated with oral health. Instead of individual identities being treated as risk factors, within intersectionality they are understood as proxies for social forces, thereby shifting the focus from individual behavior and blame to roles of power, privilege, and social accountability (Green et al. 2017).

The term “intersectionality” was coined by Black feminist legal scholar Kimberlé Crenshaw in 1989 to challenge the dominant White privileged perspective that viewed race and gender in isolation from each other. She focused on how these and other discriminations intersect in the lives and experiences of Black women in the United States (Crenshaw 1989; Moradi and Grzanka 2017)

Else-Quest and Hyde (2016) suggest that intersectionality research needs to consider 3 key elements: 1) the experiences and realities of individuals focusing on their multiple social identities, 2) a critical examination of power and inequality, and 3) understanding individual and social contexts as fluid and dynamic. Bowleg (2021, p. 88) cautions about “flattening,” a term “to describe how intersectionality, as it becomes mainstream, is being depoliticized and stripped of its attention to power, social justice, and praxis.”

Seeking to understand the complexity of older adults’ lived experience is reflected in the growing use of an intersectionality lens in a range of research topics, including agism in the COVID-19 pandemic (Rueda 2021) and health-related quality of life in people aging with HIV in China, Europe, and Latin America (Hsieh et al. 2022).

Bringing an intersectionality approach to oral health recognizes relations of power, inequity, oppression, and social exclusion when addressing the hard-to-resolve problem of poor oral health in older adults (Yuval-Davis 2016; Beaton et al. 2020). There has been limited application of intersectionality in oral health research. In 2 studies, Schuch et al. (2021) and Jamieson et al. (2023) considered intersectionality in relation to racial discrimination and oral health in Australia in their quantitative analysis. Recent literature suggests substantial potential using intersectionality in research related to aging and oral health (Cuesta and Rämgård 2016; Beaton et al. 2020; Muirhead et al. 2020; Macdonald et al. 2022; Ramos-Gomez and Kinsler 2022). This approach has the potential to highlight social injustice and build capacity in oral health care providers to improve oral health outcomes in adults across all social indices (Rosenthal 2016).

This is an important caution against uncritically accepting models such as the social determinants of health, particularly when incorporating older approaches, such as focusing on the individual devoid of one’s social context. Instead, new theoretical models can challenge our thinking around causation and deepen our inquiry to better understand and respond to relations of power that can impede or facilitate the need for structural change.

## Opportunities to Reframe Oral Health in Older Adults by Using Ecosocial Theory and Intersectionality

Current approaches have failed to resolve the wicked problem of poor oral health in older adults. Bringing ecosocial theory together with intersectionality offers a new way to think about oral health in older adults.

To begin, this combination will help address an overemphasis on individual behaviors rather than structural determinants for exploring health inequities and injustice (Laverack 2012; Baum and Fisher 2014; Krieger 2014) across older populations. More explicitly, it can deepen our inquiry to think about how race, socioeconomic status, and gender intersect with age and influence oral health.

In a recent article, Krieger (2020) recommended expanding structural measures, considering exposure in relation to the life course and historical context, factoring in measures of anti-*isms*, and “developing terrestrially grounded measures that can reveal links between the structural drivers of unjust isms and their toll on environmental degradation, climate change, and health inequities” (p. 37).

We have limited knowledge of meanings and experiences associated with oral health as people age and how experiences and perceptions may vary. For example, in Australia, when social groups from culturally and linguistically diverse backgrounds different from the Anglo-Australian norm are researched, understanding their similarities and differences can help guide a more targeted approach to policy and practice to better meet the oral health needs of these populations (and potentially apply to other health issues; Charbonneau et al. 2014; Adebayo et al. 2016).

Rather than focusing on individual oral health pathology, addressing the complexities embedded in how people experience oral health will better respond to this wicked problem. Intersectionality provides layered and textured analyses to examine how social, cultural, and political elements intersect and inform perceptions and experiences related to oral health in minority groups and affect health outcomes (Muirhead et al. 2020). For example, social determinants related to race, culture, gender, socioeconomic status, and age are seen, not as isolated, but as interrelated determinants that can reproduce and compound vulnerability and discrimination that negatively affect health outcomes (Freeman et al. 2020; Muirhead et al. 2020).

## How This Might Translate

The epidemiologic risk factor approach focusing on studying the determinants of disease across homogenized populations has been common in public health (Susser 1996; Green et al. 2017). Research within an ecosocial and intersectionality framework moves away from this approach to consider the specificities of context and how power structures affect health outcomes rather than inequalities being the result of independent risk factors (Green et al. 2017; Merz et al. 2021).

While use of ecosocial theory has been established for some time in social epidemiology, such use of theory can be enriched by intersectionality (Merz et al. 2021). Until now, oral health research has largely ignored how the interrelationship among social identities, including gender, race, age, socioeconomic status, and sexuality, affects health outcomes (Muirhead et al. 2020). An obvious approach is to use mixed methods where ecosocial theory, largely epidemiologic, and intersectionality, largely qualitative, can complement each other and add depth to the research findings.

An ecosocial and intersectionality approach allows us to consider how upstream social determinants (e.g., racism and sexism) intersect and can discriminate in ways that shape the opportunities and lived experience of individuals with multidimensional social identities (Green et al. 2017). Ecosocial theory and intersectionality challenge our thinking and approach in synergistic ways. However, according to Merz et al (2021, p. 131), “it is plausible that the study of multiple interlocking systems of power does not align well with epistemological and methodological premises of conventional, risk factor-oriented epidemiology.”

## Discussion

Despite international research over many years investigating oral health among older adults, it remains a largely intractable problem.(Thomson and Ma 2014; GBD 2017 Oral Disorders Collaborators et al. 2020). Ecosocial theory and intersectionality provide useful and holistic frameworks for addressing issues in health and now in oral health (Freeman 2020; Muirhead et al. 2020). These 2 approaches are synergistic, offering important opportunities to reconceptualize research and practice.

As noted, ecosocial theory is long established in social epidemiology, and intersectionality has largely been used within social science. Bringing the 2 together for oral health in aged care makes a strong case for more interdisciplinary mixed methods research to properly explore this complexity.

Windsong (2018) argues that we need to consider intersectionality in data collection and interviews to understand, not just how determinants and *-isms* intersect, but also how they affect perceptions and experiences of oral health in aging. It is important therefore to engage consumers’ voices at the center of the research process (Benzian et al. 2015), as this can disrupt power structures focusing on expert-based research processes that exclude consumer voices (Farr 2018). Instead, privileging their voices allows consumers to define their problems and how best to address them and to identify what matters to them based on their own knowledge and experiences (Boyle and Harris 2009; Muirhead et al. 2020). This approach alludes to Kimberlé Crenshaw’s life experience as an African American feminist who challenged the then-dominant White norms of feminism and highlighted the importance of intersectionality.

The reductive approaches to disadvantage to date have produced a focus on individual responsibility and singular layers of disadvantage, rather than addressing the larger and underlying complex issues around power and marginalization and their impact on health outcomes. There has been limited application of intersectionality in oral health research but recent research suggests substantial potential (Freeman et al. 2020; Muirhead et al. 2020), with application of this framework enhancing opportunities for social justice (Rosenthal 2016). The consideration of ecosocial theory adds to this insight and opportunity: “Given their explicitly political orientation, intersectionality and eco-social theory may well contribute to the reinvigoration of the focus on what Stonington et al. (2018, p. 1958) have called the ‘structural determinants of the social determinants of health,’ the structural causes of observed health inequities grounded in political economy, institutional discrimination, or transgenerational trauma’” (Merz et al. 2021, p. 127).

Using these approaches in research in older adult oral health would ideally raise understanding of the multiple impacts of marginalization, racism, and sexism on the oral health of older people, including their capacity to access care. Appropriate attention on structural issues and movement away from the focus on individual (and blame) can draw attention to where the problem lies and how best to address it.

Oral care should be closely aligned with culturally appropriate and community-centered primary care that highlights the importance of centring the consumer voice in decisions related to their care, enhancing understanding that every older person has a different perspective (Freeman 2020). Some of the approaches to care (e.g., interdisciplinary) then fit in our understanding from research. Having multiple professionals engaged in oral care may reduce risk of marginalization and assist in support for the consumer.

Given slow progress in improving oral health in older adults, new approaches are required that better understand and interpret the complexity of context and influences and act on power imbalances and their negative effect on health outcomes. Ecosocial theory and intersectionality offer compatible frameworks to guide teaching, research, policy responses, and practice. Understanding how power relations produce and reproduce marginalization and how to best redress resultant inequities is imperative if oral health outcomes in this cohort are to improve. This insight should inform research, professional training, policy, and provision of dental care and increase the focus on equity, prevention, interdisciplinary care, and effective use of innovative technology.

## Author Contributions

L. Slack-Smith, contributed to conception and design, data acquisition, analysis, and interpretation, drafted and critically revised the manuscript; T. Ng, contributed to design, data acquisition, analysis, and interpretation, drafted and critically revised the manuscript; M.E. Macdonald, contributed to conception and design, data analysis and interpretation, drafted and critically revised the manuscript; A. Durey, contributed to conception and design, data interpretation, drafted and critically revised the manuscript. All authors critically revised the draft and approved the final version.
